# The Expression Profiles of the *Salvia miltiorrhiza* *3-Hydroxy-3-methylglutaryl-coenzyme A Reductase 4* Gene and Its Influence on the Biosynthesis of Tanshinones

**DOI:** 10.3390/molecules27144354

**Published:** 2022-07-07

**Authors:** Małgorzata Majewska, Piotr Szymczyk, Jan Gomulski, Agnieszka Jeleń, Renata Grąbkowska, Ewa Balcerczak, Łukasz Kuźma

**Affiliations:** 1 Department of Biology and Pharmaceutical Botany, Medical University of Lodz, Muszyńskiego 1, 90-151 Lodz, Poland; piotr.szymczyk@umed.lodz.pl (P.S.); jan.gomulski@umed.lodz.pl (J.G.); renata.grabkowska@umed.lodz.pl (R.G.); 2 Laboratory of Molecular Diagnostics and Pharmacogenomics, Department of Pharmaceutical Biochemistry and Molecular Diagnostics, Medical University of Lodz, Muszyńskiego 1, 90-151 Lodz, Poland; agnieszka.jelen@umed.lodz.pl (A.J.); ewa.balcerczak@umed.lodz.pl (E.B.)

**Keywords:** *Salvia miltiorrhiza*, *HMGR4*, expression, overexpression, tanshinone, GA_3_, IAA, SA

## Abstract

*Salvia miltiorrhiza* is a medicinal plant that synthesises biologically-active tanshinones with numerous therapeutic properties. An important rate-limiting enzyme in the biosynthesis of their precursors is 3-hydroxy-3-methylglutaryl-coenzyme A reductase (HMGR). This study presents the organ-specific expression profile of the *S. miltiorrhiza HMGR4* gene and its sensitivity to potential regulators, viz. gibberellic acid (GA_3_), indole-3-acetic acid (IAA) and salicylic acid (SA). In addition, it demonstrates the importance of the *HMGR4* gene, the hormone used, the plant organ, and the culture environment for the biosynthesis of tanshinones. *HMGR4* overexpression was found to significantly boost the accumulation of dihydrotanshinone I (DHTI), cryptotanshinone (CT), tanshinone I (TI) and tanshinone IIA (TIIA) in roots by 0.44 to 5.39 mg/g dry weight (DW), as well as TIIA in stems and leaves. *S. miltiorrhiza* roots cultivated in soil demonstrated higher concentrations of the examined metabolites than those grown in vitro. GA_3_ caused a considerable increase in the quantity of CT (by 794.2 µg/g DW) and TIIA (by 88.1 µg/g DW) in roots. In turn, IAA significantly inhibited the biosynthesis of the studied tanshinones in root material.

## 1. Introduction

*Salvia miltiorrhiza* Bunge, also known as Chinese sage or Red sage, is one of the basic elements of traditional Chinese medicine used in treating diverse conditions, such as cardiovascular diseases, menstrual disorders and insomnia [1,2]. The medical properties of this plant result from the biosynthesis of various bioactive compounds, including tanshinones. Recent research indicates that tanshinones provide cardiovascular protection [3], regulate metabolic functions [4], and possess a range of anticancer [5], neuroprotective [6], anti-inflammatory [7], antioxidant [8], phytoestrogenic [9], antiosteoporotic [10], antibacterial [11] and anti-aggregation [12] properties.

Among the several dozen tanshinones isolated from *S. miltiorrhiza* so far [13], the most studied are dihydrotanshinone I (DHTI), cryptotanshinone (CT), tanshinone I (TI) and tanshinone IIA (TIIA). The starting point for the production of the tanshinone diterpene backbone is the synthesis of isopentenyl pyrophosphate (IPP) and dimethylallyl pyrophosphate (DMAPP) precursors through the mevalonate (MVA) and methylerythritol phosphate (MEP) pathways [14]. The key rate-limiting enzyme in the MVA pathway, catalysing the conversion of 3-hydroxy-3-methylglutaryl-coenzyme A (HMG-CoA) to MVA, is HMG-CoA reductase (HMGR) [14]. A recent search found five sequences of *S. miltiorrhiza HMGR* genes (*HMGR* to *HMGR4*) currently deposited in the GenBank database [15,16,17]. Among them is *HMGR4*, which, unlike other genes, has not been extensively studied, and its importance in the biosynthesis of tanshinones has not been investigated.

Whereas the annual consumption of *S. miltiorrhiza* in China exceeds 16 million kg, the low concentration of tanshinones in plant material and limited arable land available for cultivation, make the meeting of growing demand more and more problematic [14]. Therefore, alternative sources of *S. miltiorrhiza* plant material, such as hairy roots or cell and callus cultures are under development [18]. These sources offer continuous biosynthesis of tanshinones, and their concentration may be boosted by the application of chemical or physical components known as elicitors [19,20,21].

Gibberellic acid (GA_3_) regulates vegetative and reproductive growth by triggering the degradation of DELLA proteins, these being master repressors of its signaling [22,23]. Initially, a bioactive hormone binds to the gibberellin-insensitive DWARF 1 (GID1) receptor and induces a conformational change in its N-terminal fragment, enabling DELLA binding. The DELLA proteins are then polyubiquitinated by E3 ubiquitin ligases such as SLEEPY1 and constitutively photomorphogenic 1 (COP1), and directed towards the destruction in 26S proteasome [24]. Removing the DELLA proteins releases repressed transcription factors (TFs), enabling gene expression regulation. Dominant TFs regulated by GA_3_ are GAI-RGA-SCR (GRAS) proteins [25]. In *S. miltiorrhiza* most of the 35 identified GRAS TFs are induced by GA_3_ [25]. It has been proven that the overexpression of *GRAS1* and *GRAS2* in *S. miltiorrhiza* hairy roots increases the accumulation of DHTI, CT, TI and TIIA [26].

In the auxin signaling pathway, the transcription of effector genes is controlled by the interaction of auxin/indole-3-acetic acid (Aux/IAA) repressor with transport inhibitor resistant 1/auxin signaling F-box (TIR1/AFB) proteins [27]. Ubiquitination of these complexes by suppressor of kinetochore protein 1 (SKP1)/cullin1/F-box (SCF) E3 ubiquitin ligase complex and subsequent proteasome-dependent degradation, enable auxin response factors (ARFs) to regulate gene transcription [28]. The proper initiation of gene expression usually requires dimerization of ARFs, that bind to closely-located TGTCGG inverted repeats and TGTCTC or TGTCGG direct repeats [29]. The complexity of auxin-dependent gene regulation in *S. miltiorrhiza* is increased by the fact that most of the 25 studied ARFs have an inhibitory effect on the transcription rate [30].

In the salicylic acid (SA) signaling route, the non-expressor of pathogenesis-related genes 1 (NPR1) acts as the master regulator of the plant response [31]. In the absence of SA, the N-terminal BTB domain of NPR1 interacts with the C-terminal transactivation domain to inhibit NPR1 transcription activity [32]. NPR1 is activated through copper-dependent binding of SA [32]. This protein lacks its own DNA-binding domain and expresses its trans-activatory function through interaction with bZIP family TFs [33]. Such SA-responsive TGACG transcription factor binding sites (TFBSs) have been found in numerous plant promoters [34,35,36]. Another group of TFs controlled by SA are WRKY; among these, WRKY1 strongly induces the genes of the tanshinone biosynthesis pathway through interaction with the W-box (T)TGAC(C/T) element [37].

This work examines the organ-specific expression pattern of the *S. miltiorrhiza HMGR4* gene and the influence of selected phytohormones (GA_3_, indole-3-acetic acid (IAA), SA) on its transcription level. These experiments were carried out on wild, in vitro-grown plants. In silico analysis of the *S. miltiorrhiza HMGR4* promoter performed with PlantPan 2.0 tool was used to select the appropriate hormones. Moreover, this study investigates the importance of the *HMGR4* gene, the hormone used, the plant organ and the growth environment for the biosynthesis of DHTI, CT, TI, TIIA and total tanshinone using transgenic *S. miltiorrhiza* plants grown in vitro and in soil. The use of hormones is aimed at modulating the *HMGR4* gene expression and thus obtaining information on the presumed role of this gene in the biosynthesis of tanshinones, as well as influencing their content.

## 2. Results

### 2.1. Organ-Specific Expression of S. miltiorrhiza HMGR4 Gene

Real-time qPCR results showed that *HMGR4* gene was expressed in all analysed *S. miltiorrhiza* organs, but with different intensities. The leaves and stems demonstrated higher levels of the *HMGR4* transcript than the reference, with R = 1.14 ± 0.08 and R = 1.05 ± 0.01, respectively; in roots, the level was lower than in the reference with R = 0.95 ± 0.07.

Due to their level of transcript and high availability of material for research, it was decided to use the leaves to study the effect of hormones on *S. miltiorrhiza HMGR4* activity.

### 2.2. Potential Regulators of S. miltiorrhiza HMGR4 Gene Expression

Sequence analysis of the *S. miltiorrhiza HMGR4* promoter using the PlantPan 2.0 tool showed the existence of 5369 potential TFBSs and 365 interacting TFs previously detected in the *Arabidopsis thaliana* model plant. The similarity score between the TFBSs found in *HMGR4* promoter and those identified in *A. thaliana* was set to 0.7–1.0. Of all the TFs detected, a large group was able to respond to hormonal agents; many of these were sensitive to GA_3_, IAA and SA (Table 1, Tables S1–S3). It is worth emphasising that these TFs also had potential binding sites in the *HMGR4* proximal promoter region (Tables S1–S3), where most functional TFBSs are believed to be located [38,39]. Therefore, it was decided to investigate the importance of these hormones on the expression of *S. miltiorrhiza HMGR4*.

### 2.3. Effect of GA_3_, IAA, SA on S. miltiorrhiza HMGR4 Gene Expression

**Figure 1:**
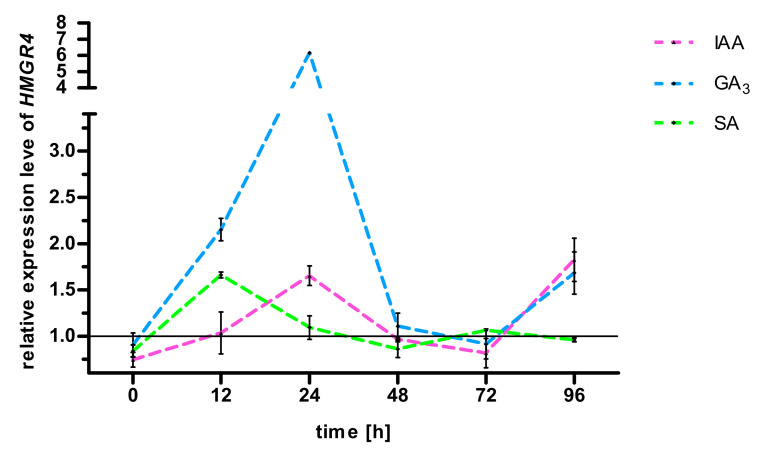
Effect of GA_3_, IAA and SA on *HMGR4* gene expression in *S. miltiorrhiza* leaves. The expression was analysed by real-time PCR. The results are presented as mean ± SD.

The hormones used in the experiment changed the expression of *HMGR4* in treated leaves compared to control (leaves not incubated with hormones) (Figure 1). At the beginning of each study, a lower *HMGR4* transcript level was observed in the test materials than in corresponding control (R < 1). Treatment with GA_3_ or IAA or SA for 12 and 24 h resulted in the stimulation of *HMGR4* expression against untreated samples (R > 1). It is worth noting that the exposure of leaves to GA_3_ resulted in an approximately 2.86-fold increase in *HMGR4* expression between 12 and 24 h, and the stimulation effect was also maintained at 48 h. From 48 h, the level of *HMGR4* transcript in the hormone-treated samples decreased compared to the control (R < 1). In the final part of the testing (72 h for SA and 96 h for GA_3_ and IAA) the level of *HMGR4* mRNA increased again in leaves incubated with hormones compared to untreated samples (R > 1).

Based on the obtained results, GA_3_ and IAA were selected for experiments determining the tanshinone content.

### 2.4. Impact of pRI201-AN-HMGR4 Transformation on S. miltiorrhiza HMGR4 Gene Expression

Higher levels of the *HMGR4* transcript were observed in all *S. miltiorrhiza* organs taken from plants transformed with the pRI201-AN-HMGR4 construct compared to control (R > 1). The R values for stems, roots and leaves, were 1.28 ± 0.19, 1.25 ± 0.14 and 1.10 ± 0.14, respectively.

### 2.5. Influence of HMGR4 Overexpression on the Biosynthesis of Tanshinones in S. miltiorrhiza

The conducted studies indicate that *S. miltiorrhiza HMGR4* overexpression had a significant influence on the quantity of measured tanshinones.

**Figure 2:**
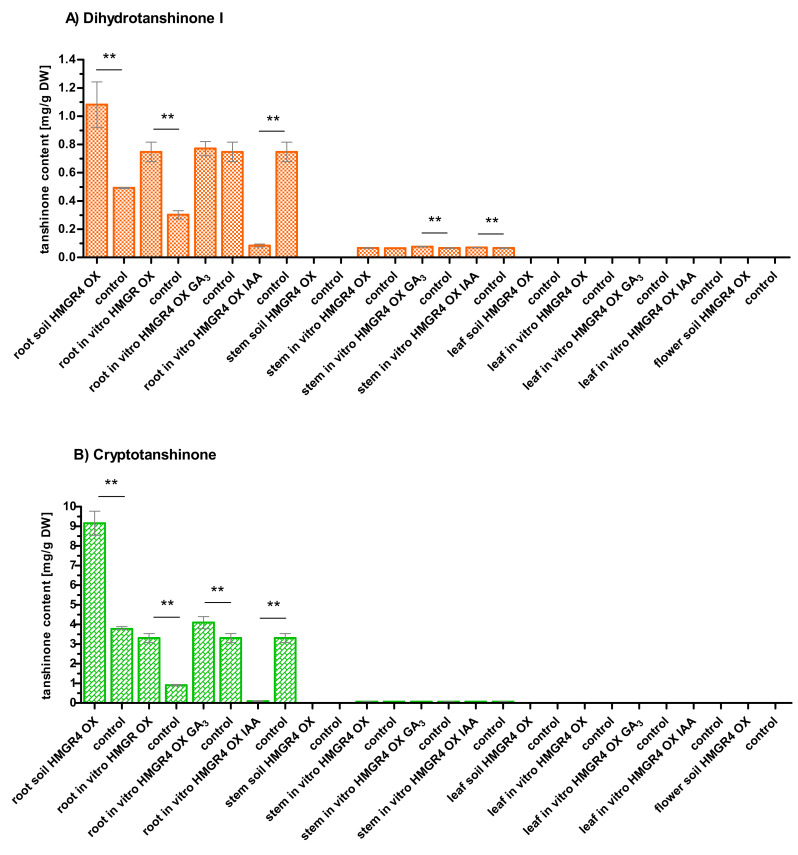

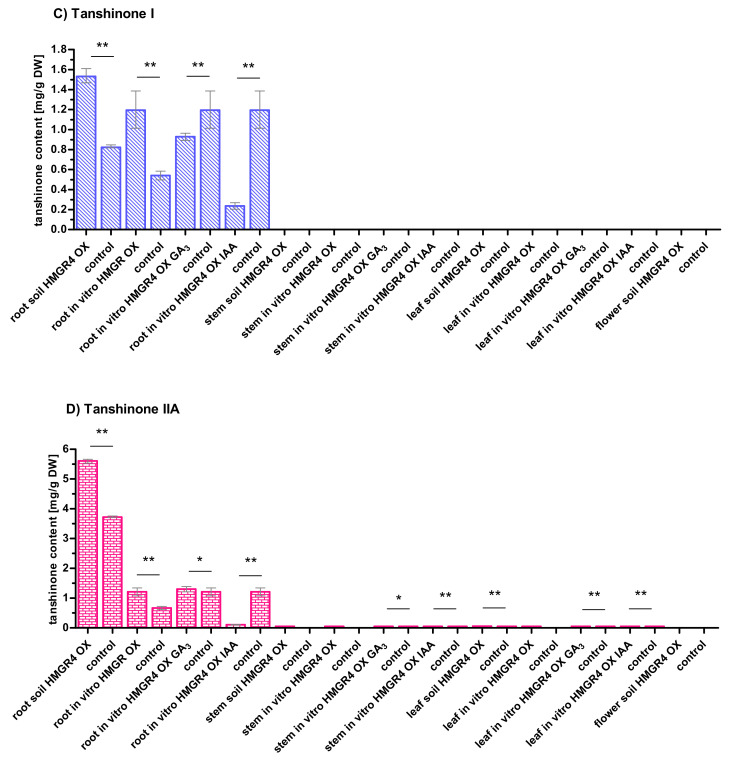

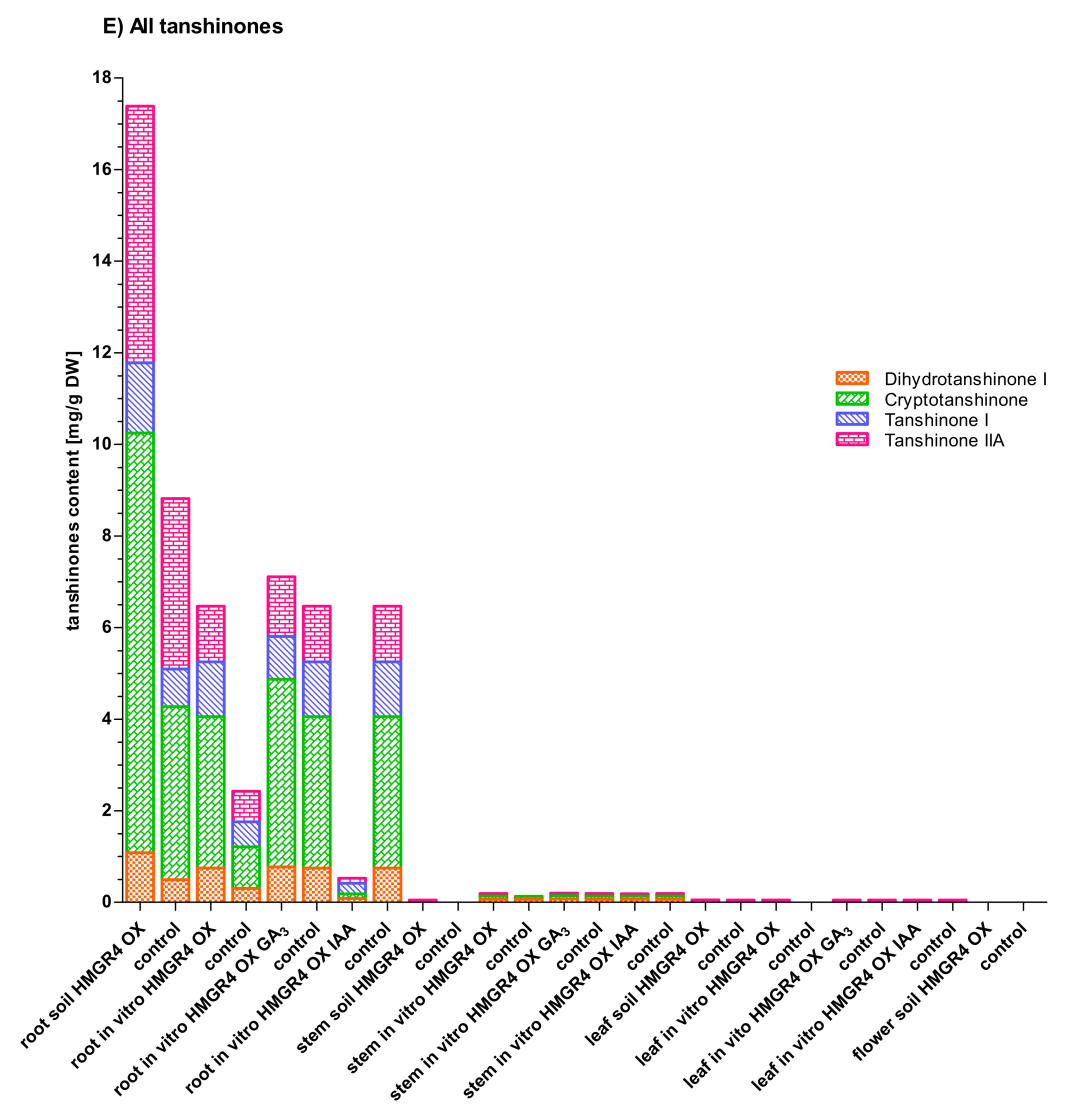
Quantitative UHPLC analysis of the content of individual tanshinones (**A**–**D**) and the total amount of all tested tanshinones (**E**) in extracts obtained from roots, stems, leaves and flowers of *S. miltiorrhiza*. A control is shown for each test sample (non-*HMGR4* overexpressing plant material or, in the case of hormone treatment, non-hormone-treated *HMGR4* overexpressing plant material). Bars are medians with first and third quartile. ** significant difference at *p* < 0.01 compared to control; * significant difference at 0.01 < *p* < 0.05 compared to control; DW, dry weight; OX, overexpression.

Roots with *HMGR4* overexpression, both soil-grown and cultivated in vitro, demonstrated significantly higher accumulation of DHTI, CT, TI, TIIA and higher total tanshinone content compared to control roots without *HMGR4* overexpression (*p* < 0.01) (Figure 2A–E). The differences described above ranged from 1.51-fold to 2.43-fold, i.e., 0.59–5.39 mg/g dry weight (DW), and from 1.82- to 3.62-fold (0.44–2.40 mg/g DW), respectively, depending on the type of tanshinone.

Clear differences in the levels of individual tanshinones were observed between roots with *HMGR4* overexpression and those without. More specifically, these values were as follows (the first value indicating soil conditions and the second in vitro): 2.43- or 3.62-fold (5.39 or 2.40 mg/g DW) for CT, 2.19- or 2.47-fold (0.59 or 0.44 mg/g DW) for DHTI, 1.86- or 2.21-fold (0.71 or 0.65 mg/g DW) for TI, 1.51- or 1.82-fold (1.88 or 0.55 mg/g DW) for TIIA, and reflected the place of cultivation.

Moreover, overexpression of *HMGR4* gene induced the quantity of TIIA to about 50 µg/g DW in in vitro and in soil-grown stems and leaves (Figure 2D).

### 2.6. Organ-Dependent Accumulation of Tanshinones in S. miltiorrhiza

Roots appeared to be the main site of accumulation of all studied metabolites in *S. miltiorrhiza*. All of the examined roots were found to contain all tested tanshinones (Figure 2A–D). CT was present at the highest levels (0.91–9.17 mg/g DW), while lower amounts were found for TIIA (0.67–5.61 mg/g DW), TI (0.54–1.53 mg/g DW) and DHI (0.30–1.08 mg/g DW) (Figure 2A–D). The quantity of the identified metabolites was highest in soil-grown roots overexpressing *HMGR4*.

Some tanshinones were detected in stems and leaves with median values ranging from 50 to 73.5 µg/g DW (Figure 2A,B,D,E). The most common tanshinone present in the tested stems and leaves was TIIA. The TIIA content was typically 104.5-fold higher (by 5.55 mg/g DW) in roots than in stems or leaves in the soil-grown plants, and 23.4-fold higher (by 1.16 mg/g DW) in in vitro roots (Figure 2D). No tanshinones were detected in flowers (Figure 2A–E). Hence, apart from slight changes in TIIA level in stems and leaves, *HMGR4* overexpression did not appear to significantly change the organ-specific pattern of accumulation of the compounds in *S. miltiorrhiza*.

### 2.7. Impact of Growth Environment on the Biosynthesis of Tanshinones in S. miltiorrhiza

The soil environment favoured a significantly higher production of all tested tanshinones in the root material compared to in vitro conditions (*p* < 0.01). This was true both in the group of roots with and without *HMGR4* overexpression, and the differences were from 1.45- to 4.62-fold (0.34–5.86 mg/g DW) and from 1.63- to 5.58-fold (0.19–3.06 mg/g DW), respectively, depending on the type of metabolite. The quantities of individual tanshinones varied considerably between the soil-grown roots and those grown in vitro. More specifically, these differences were as follows (first value = *HMGR4* overexpression; the second value = without): 4.62- or 5.58-fold (4.39 or 3.06 mg/g DW) for TIIA, 2.77- or 4.14-fold (5.86 or 2.87 mg/g DW) for CT, 1.45- or 1.63-fold (0.34 or 0.19 mg/g DW) for DHTI, 1.28- or 1.52-fold (0.34 or 0.28 mg/g DW) for TI. It is worth noting that the in vitro roots with *HMGR4* overexpression demonstrated 1.51-fold higher DHTI (0.25 mg/g DW) and 1.45-fold higher TI (0.37 mg/g DW) than the soil-grown roots without overexpression.

In leaf material, the content of TIIA was significantly higher in soil than in in vitro conditions (*p* = 0.0000), amounting to 50 or 56.8 µg/g DW, depending on *HMGR4* overexpression status.

DHTI and CT were detected in stems grown in vitro but not in stems grown in soil (Figure 2A,B). Median levels were 66.8 or 65.9 µg/g DW for DHTI, and 72 or 67.3 µg/g DW for CT, depending on the presence or absence of *HMGR4* overexpression.

### 2.8. Effect of GA_3_ and IAA on the Biosynthesis of Tanshinones in S. miltiorrhiza

The addition of GA_3_ to *S. miltiorrhiza* in vitro root culture significantly increased CT, TIIA and total tanshinone levels in comparison to untreated roots (*p* = 0.0000, *p* = 0.0404, *p* = 0.0404, respectively) (Figure 2B,D). The observed increases were 1.24-fold (0.79 mg/g DW) for CT and 1.07-fold (88.1 µg/g DW) for TIIA. Treatment had no effect on DHTI and significantly decreased the amount of TI by 1.29-fold (0.27 mg/g DW) (*p* = 0.0000) (Figure 2A,C).

In vitro cultivation of stems grown in the presence of GA_3_ showed a significant 1.15-fold (9.9 µg/g DW) rise in DHTI (*p* = 0.0000) and a significant 1.04-fold (1.9 µg/g DW) reduction in TIIA (*p* = 0.0235) compared to untreated controls (Figure 2A,D). However, in vitro cultivation of leaves with GA_3_ resulted in a significant 1.02-fold (1.2 µg/g DW) increase in TIIA compared to control (*p* = 0.0000) (Figure 2D).

The use of IAA resulted in a significant decrease in the content of all tested tanshinones in in vitro root culture compared to untreated roots (*p* = 0.0000) (Figure 2A–D): 34.06-fold (3.21 mg/g DW) for CT, 11.49-fold (1.11 mg/g DW) for TIIA, 8.84-fold (0.66 mg/g DW) for DHTI and 5.05-fold (0.96 mg/g DW) for TI.

IAA treatment only appeared to have a slight influence on the quantity of tanshinones in stems and leaves: a significant 1.05-fold (3.2 µg/g DW) rise in DHTI and a significant 1.05-fold (2.3 µg/g DW) fall in TIIA were observed in stems compared to control (*p* = 0.0000) (Figure 2A,D), while a significant 1.01-fold (0.7 µg/g DW) increase in TIIA was noted in leaves relative to control (*p* = 0.0001) (Figure 2D).

## 3. Discussion

This work analyses the expression profiles of the *S. miltiorrhiza HMGR4* gene and its influence on the biosynthesis of tanshinones.

Previous studies have shown that *S. miltiorrhiza HMGR* genes are expressed in the roots, stems and leaves, but with different intensities in each organ. *HMGR* showed the strongest activity in roots, and weaker in stems and leaves [17]. The level of the *HMGR2* transcript was about four-fold higher in leaves than in stems, and about two-fold higher in stems than in roots [15]. *HMGR3* was vigorously expressed in stems and root steles, and to a much greater degree than in root cortices and leaves [16]. *HMGR4* activity was the highest in flowers, lower in stems and leaves, and lowest in root steles and root cortices [16]. In the present study, a higher level of *HMGR4* mRNA was noted in leaves and stems than in the control; however, this was not observed in roots. Previous transcriptomic analyses have indicated that within the *S. miltiorrhiza* root, the strongest expression of *HMGR4* occurred in xylem [40].

The *S. miltiorrhiza HMGR4* gene showed a biphasic response to GA_3_ treatment. After initial stimulation of its expression relative to the control at 12, 24 and 48 h, it then decreased and subsequently increased at 96 h (Figure 1). It has been found that 2.89 µM GA_3_ has a similar influence on the *S. miltiorrhiza HMGR2* gene; however, in this case, *HMGR2* expression increased compared to control at 12 h, followed by a fall and a second increase at 72 and 96 h [41]. Elsewhere, stimulation with 400 µM GA_3_ resulted in an initial rise in *Malus domestica HMGR1* transcripts against control until four hours, followed by a decrease at six h [42]. We hypothesise that stimulation of *S. miltiorrhiza HMGR4* gene expression by GA_3_ and subsequent enzyme production could activate the next stages of the MVA pathway and the production of mediators necessary for the biosynthesis of endogenous gibberellins, such as ent-kaurene [43]. The newly-produced endogenous GA_3_ could stimulate the *HMGR4* transcription which decreased as a result of metabolising the exogenous hormone. However, this hypothesis needs to be verified by monitoring endogenous GA_3_ levels during the course of an experiment.

The impact of IAA on *S. miltiorrhiza HMGR4* expression was very similar to that induced by GA_3_ (Figure 1). Although the effect of IAA on plant *HMGR* genes has not been widely studied, we have noticed some similarities in our results with previous research. IAA at a final concentration of 100 µM first raised the level of *M. domestica HMGR4* transcripts relative to control, and then lowered them [42]. The biphasic effect, which we observed in our experiment, may result from the stimulation of various TFs, some of which increase expression of the gene, while others reduce it.

The use of SA caused a rise in *HMGR4* expression at 12, 24 and 72 h and a fall at 48 and 96 h in relation to untreated material (Figure 1). A similar effect was observed for 10 mM SA against *HMGR3* in *Ginkgo biloba* leaves; however, in contrast to our present findings, the level of *HMGR3* mRNA rose against control values in the final phase of the study (96 and 120 h) [44]. Elsewhere, SA treatment was found to result in continually elevated *HMGR* transcript levels versus untreated controls in *S. miltiorrhiza* hairy roots throughout the experiment [20] and *Salvia przewalskii* hairy roots [45]. A maximum three-fold increase in *HMGR* expression was noted after 36 h of stimulation [20], and an eight-fold rise after six days [45].

The present study is the first investigation of the role of *HMGR4* in the biosynthesis of tanshinones in *S. miltiorrhiza*. Overexpression of this gene resulted in a significant increase in DHTI, CT, TI and TIIA content: by 1.51- to 2.43-fold (0.59–5.39 mg/g DW) in soil-grown roots, and by 1.82- to 3.62-fold (0.44–2.40 mg/g DW) in in vitro roots (Figure 2A–D). Of all tanshinones tested, CT showed the highest rise relative to control: 2.43-fold (5.39 mg/g DW) for roots grown in soil and 3.62-fold (2.40 mg/g DW) for in vitro roots. The results are in agreement with data received for other *S. miltiorrhiza* HMGR enzymes. Kai et al. reported that overexpression of the *HMGR* gene led to an increase in CT, TI, TIIA quantity in hairy root culture ranging from 1.17- to 3.19-fold (0.844–1.515 mg/g DW) compared to control [46]. As in our research, CT showed the highest rise in all seven transgenic lines tested. In another study, *HMGR2* overexpression significantly enhanced the amount of DHTI, CT, TI, TIIA by 1.23- to 2.46-fold (0.99–3.16 mg/L) at day 40 of root culture relative to control [15]. In the experiment, CT demonstrated the greatest increase, i.e., by 2.46-fold (3.16 mg/L).

Our results indicate that tanshinone accumulation in *S. miltiorrhiza* was organ-dependent, with roots as the primary storage place for DHTI, CT, TI, TIIA (Figure 2E). Li et al. specifically indicate the root periderm of *S. miltiorrhiza* as the main site of accumulation of all tested tanshinones, viz. DHTI, CT, TI, TIIA, Tanshinone IIB, Dehydrotanshinone IIA, Dashenxinkun B, Trijuganone A, Trijuganone C; the inner layer of the roots and the outer part of stems contained much smaller amounts [47]. Subsequent research also pointed to *S. miltiorrhiza* root periderm as the main storage place for TIIA, although traces were also detected in root phloem [40]. In addition, transcriptomic analyses of the MVA and MEP pathway genes and other enzymes leading to the production of tanshinones indicated that the strongest expression of most of the tested genes (*AACT1* to *AACT6*, *HMGS2*, *HMGR1*, *HMGR2*, *MK*, *PMK*, *MDC1*, *MDC2*, *IPI1*, *GGPPS3*, *DXS2*, *DXS4*, *DXR*, *MCT*, *CMK*, *MDS*, *HDS*, *HDR1* to *HDR3*, *CPS1*, *CPS5*, *KSL1*, *KSL7*, *KSL8*, *CYP76AH1*) occurred in the periderm of *S. miltiorrhiza* roots [40]. Hence, the root periderm layer appears to be not only the main storage site, but also the main place of biosynthesis of tanshinones. The examined stems turned out to be a better source of the metabolites than leaves, but their content was quite low (several dozen µg/g DW) (Figure 2E). These results are in line with previously-performed studies [48]. Organ-specific accumulation and production of tanshinones may result from the existence of various mechanisms regulating the activity of enzymes involved in the biosynthesis of these compounds [49].

Our findings indicate that soil cultivation favoured 1.28- to 5.58-fold (0.19–5.86 mg/g DW) higher production of DHTI, CT, TI and TIIA in roots and 1.12-fold (6.2 µg/g DW) greater production of TIIA in leaves compared to in vitro conditions. This may be due to the community of microorganisms naturally present in the rhizosphere, phyllosphere and endosphere; it is possible that these may affect the biosynthesis of metabolites [50,51]. According to Yan et al., the endophytic bacteria *Pseudomonas brassicacearum* subsp. *neoauraniaca* raised the activity of HMGR and DXS enzymes by 2.1- and 4.2-fold, respectively, in *S. miltiorrhiza* hairy root culture. This resulted in a significant increase in the content of all tanshinones tested, with particular gains found for DHTI (19.2-fold) CT (11.3-fold) and total tanshinones (3.7-fold) compared to controls [52]. In addition, the polysaccharide fraction isolated from rhizobacterium *Bacillus cereus* stimulated the accumulation of tanshinones in *S. miltiorrhiza* root culture by about seven-fold (1.59 vs. 0.19 mg/g DW) compared to control [53]. Another potential reason for the lower in vitro yields of tanshinones may be changes occurring in the morphology, anatomy and physiology of plants during in vitro cultivation [54,55].

Additionally, our findings provide further information about the influence of hormones on the biosynthesis of tanshinones in *S. miltiorrhiza*. GA_3_ stimulated CT and TIIA production, but had no significant effect on DHTI content and decreased TI in in vitro root culture compared to untreated controls (Figure 2A–D). We hypothesize that the presence of GA_3_ may strongly induce the expression of some key enzyme/-s involved in the terminal stage of CT biosynthesis. This could be the reason for the higher TIIA content which arises from CT; however, as GA_3_ may not have a similar effect on DHTI production, the resulting TI does not rise, and may even fall [14]. GA_3_ has been found to increase DHTI, CT, TI and TIIA levels in most *GRAS3*-overexpressing *S. miltiorrhiza* hairy root culture lines and in untransformed controls [56]; however, these results cannot be directly compared to ours, as the experiment used a 34.6-fold higher concentration of the hormone (100 µM) and a much shorter incubation time with GA_3_, of only six days. The second hormone used, IAA, significantly reduced the accumulation of CT by 34.06-fold (3.21 mg/g DW), TIIA by 11.49-fold (1.11 mg/g DW), DHTI by 8.84-fold (0.66 mg/g DW) and TI by 5.05-fold (0.96 mg/g DW) in an in vitro root culture versus control (Figure 2A–D). Reduced CT, TI and TIIA synthesis was also observed in *S. miltiorrhiza* hairy roots treated with 5.71 µM IAA: 1.61-fold decrease (82 µg/g DW) for TI, 1.50-fold decrease (125 µg/g DW) for CT, and 1.24-fold decrease (23 µg/g DW) for TIIA, compared to control [57].

## 4. Materials and Methods

### 4.1. Establishment of S. miltiorrhiza Culture and Treatments

*S. miltiorrhiza* plants were cultivated from seeds provided by the Garden of Medicinal Plants of the Medical University of Lodz. To establish in vitro plant cultures, the seeds were surface sterilised utilising 70% ethanol for 1 min and subsequent 1% sodium hypochlorite solution for 5 min, and then rinsed three times with sterile distilled water for 5 min. The seeds were thereafter transferred aseptically onto Murashige and Skoog (MS) basal medium [58] with 3% sucrose (Chempur, Piekary Śląskie, Poland) and 0.65% agar (Sigma-Aldrich, Saint Louis, MO, USA) and a final pH of 5.7. Germination was carried out in the dark at 26 ± 2 °C. After germination, aerial parts of *S. miltiorrhiza* were grown in solid MS medium at 26 ± 2 °C under 16/8 h (light/dark) photoperiod at a cool fluorescent light with intensity of 40 μmol m*^−^*^2^ s*^−^*^1^. Roots were cultivated in the dark at 26 ± 2 °C in Gamborg B5 liquid medium [59] agitated at 70 rpm. Subcultures were carried out every five weeks.

Five-week-old leaves, stems and roots, grown as described above, were used to study organ-specific expression of the *HMGR4* gene.

The effect of hormones on *HMGR4* activity was determined in five-week-old leaves. *S. miltiorrhiza* plants were incubated in sterile distilled water containing 1 mg/L (2.89 µM) GA_3_ or 0.5 mg/L (2.85 µM) IAA or 20 mg/L (144.80 µM) SA and 0.01% non-ionic detergent Triton X-100 (Sigma-Aldrich, Saint Louis, MO, USA). Plants treated with sterile distilled water supplemented with 0.01% Triton X-100 were used as controls. Samples were collected after 0, 12, 24, 48, 72 and 96 h.

### 4.2. Selection of Potential Regulators of S. miltiorrhiza HMGR4 Gene Expression

The *S. miltiorrhiza HMGR4* promoter sequence deposited in GenBank under accession number KT921337.1 was scanned with PlantPan 2.0 tool (http://plantpan2.itps.ncku.edu.tw/, accessed on 5 June 2021) for TFBSs and interacting TFs [60]. UniProt database (https://www.uniprot.org/, accessed on 5 June 2021) was used to acquire information on received TFs [61].

### 4.3. Preparation of pRI201-AN-HMGR4 Overexpression Construct

**Figure 3:**
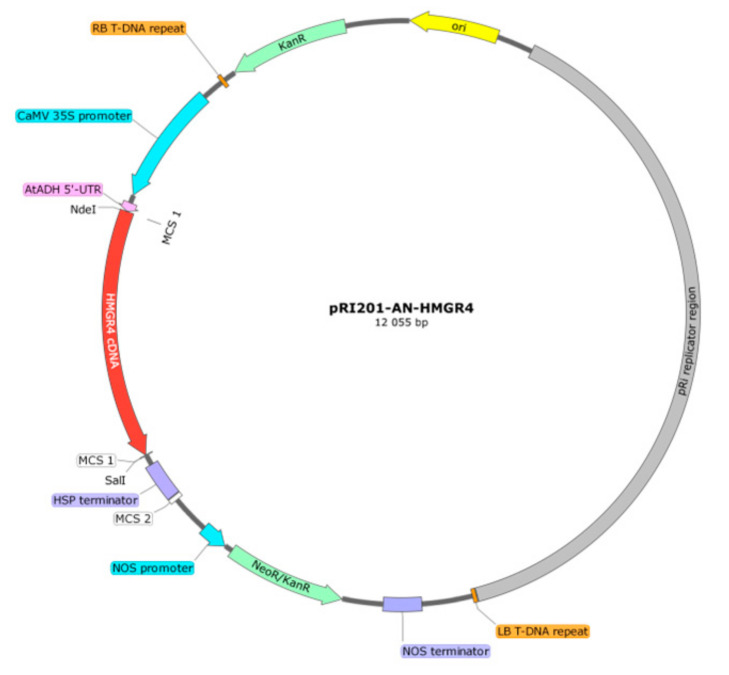
Map of the expression construct pRI201-AN-HMGR4.

The *S. miltiorrhiza HMGR4* coding sequence (1653 bp) was synthesised on the basis of JN831103.1 sequence and inserted into a pUC57 vector (Gene Universal Inc., Newark, DE, USA). The correctness of the insert was determined by double-strand Sanger sequencing. Afterwards, the *HMGR4* insert was excised from pUC57 and inserted into a pRI201-AN binary expression vector (Takara Bio Inc., Kusatsu, Japan) at NdeI/SalI sites of MCS1 (Eurofins Genomics, Ebersberg, Germany). *HMGR4* gene overexpression was driven by the strong and constitutive promoter of Cauliflower Mosaic Virus 35S (CaMV), which facilitates high levels of RNA transcription in a wide variety of plants. Analysis of the *HMGR4* sequence and flanking regions was performed by double-strand Sanger sequencing. A map of the prepared pRI201-AN-HMGR4 construct is presented in Figure 3.

### 4.4. Transformation, Selection, Regeneration and Treatments of S. miltiorrhiza Culture

*Agrobacterium tumefaciens* (*Rhizobium radiobacter*) GV2260 (C58C1Rif^R^ with pGV2260) competent cells were transformed with the pRI201-AN-HMGR4 construct or the empty pRI201-AN vector using the freeze/thaw method [62]. The transformed bacteria were firstly grown for 84 h at 26 °C on solid selective YEB medium containing 50 mg/L kanamycin, 100 mg/L carbenicillin and 30 mg/L rifampicin (Chem-Impex International, Wood Dale, IL, USA) and then on liquid selective YEB medium with shaking at 140 rpm until OD_600_ reached 0.4–0.8. To confirm the transformation, plasmid DNA was isolated by alkaline lysis and extracted with a phenol/chloroform/isoamyl alcohol mixture [63]; this was then subjected to PCR amplification using GoTaq Hot Start Green Master Mix (Promega, Madison, WI, USA) and Kanamycin primers (Table 2). The PCR reactions were carried out in an MJ Mini Personal Thermal Cycler (Bio-Rad, Hercules, CA, USA) with the following parameters: initial denaturation (95 °C, 5 min), denaturation (95 °C, 45 s), primer annealing (60 °C, 30 s), extension (72 °C, 30 s), final extension (72 °C, 5 min). In total, 40 PCR cycles were conducted. The obtained products were separated by 2% agarose gel electrophoresis.

To induce virulence, bacterial cultures with confirmed transformation were collected by centrifugation and resuspended to OD_600_ = 0.1 in sterile induction medium, i.e., liquid MS medium supplemented with 100 µM acetosyringone (Sigma-Aldrich, Saint Louis, MO, USA), and then agitated on a rotary shaker at 140 rpm for five hours at 26 °C [64].

**Figure 4:**
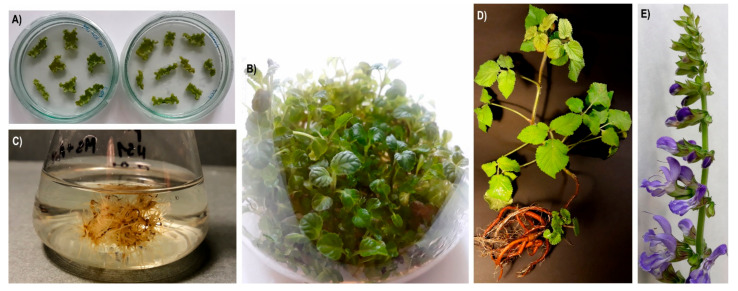
Cultures of *S. miltiorrhiza* at various stages of transformation, regeneration and growth. (**A**) callus resistant to selection antibiotics after two weeks of growth, (**B**) shoots regenerated from callus three months after the experiment began, (**C**) culture of roots transformed with the pRI201-AN-HMGR4 construct, (**D**) plant transformed with the pRI201-AN-HMGR4 construct after several months of growing in soil, (**E**) flowering of the plant transformed with the pRI201-AN-HMGR4 construct.

Three-month-old leaves of *S. miltiorrhiza* grown in pots were surface sterilised using the same protocol described earlier for the seeds; however, 0.8% sodium hypochlorite solution was applied. Preparation, infection of leaves and co-cultivation were performed according to Dandekar and Fisk with some modifications [64]. The composition of the induction medium was as mentioned above. Co-cultivation solid MS medium was supplemented with 1 mg/L 6-benzylaminopurine (BAP), 0.2 mg/L 1-naphthaleneacetic acid (NAA) and 100 µM acetosyringone (Sigma-Aldrich, Saint Louis, MO, USA). Overall regeneration frequency, non-transgenic regeneration under selection and non-transgenic controls were included in the research. After 72 h of incubation, leaf discs were transferred every two weeks onto fresh *A. tumefaciens* (*R. radiobacter*) killing medium, i.e., solid MS medium with 1 mg/L BAP, 0.2 mg/L NAA and 250 mg/L cefuroxime. After another six weeks, the obtained calluses were moved onto solid MS medium supplemented with 0.5 mg/L BAP, 0.2 mg/L IAA and 250 mg/L cefuroxime. In the following weeks, cefuroxime was gradually phased out and the selection antibiotic kanamycin (Biological Industries, Kibbutz Beit-Haemek, Israel) was introduced (10–50 mg/L). The aerial parts of the *S. miltiorrhiza* transformants and controls were cultivated in solid MS medium at 26 ± 2 °C under 16/8 h (light/dark) photoperiod using a cool fluorescent light with intensity of 40 μmol m^−2^ s^−1^ and their roots in the dark in liquid Gamborg B5 medium agitated at 70 rpm. Subcultures were carried out every five weeks. Additionally, in order to compare the influence of different growth environments on the biosynthesis of tanshinones, the transformants and control were transferred from in vitro cultures to pots containing sterile composite soil. The plants were covered with a transparent glass jar for three weeks and grown at 26 ± 2 °C under natural light. Figure 4 shows *S. miltiorrhiza* cultures at various stages of the experiment.

The transformation of *S. miltiorrhiza* plants was confirmed by PCR analysis of genomic DNA isolated from five-week-old leaves using Isolate II Plant DNA kit (Bioline, Taunton, MA, USA) according to the manufacturer’s instructions, with the use of GoTaq Hot Start Green Master Mix (Promega, Madison, WI, USA) and Kanamycin primers (Table 2). The concentration and purity of the DNA were assessed based on A_260_/A_280_ and A_260_/A_230_ ratios using a Nanophotometer P300 (Implen, Munich, Germany). PCR reaction parameters were as mentioned above. The obtained products were separated via 2% agarose gel electrophoresis.

The effect of the pRI201-AN-HMGR4 construct or the empty pRI201-AN vector (control) on *S. miltiorrhiza HMGR4* expression was investigated in five-week-old leaves, stems and roots.

The importance of the *HMGR4* gene for tanshinone biosynthesis was assessed in five-week-old *S. miltiorrhiza* roots, leaves, and stems growing in soil and in vitro and overexpressing *HMGR4* relative to plant material that did not overexpress *HMGR4*.

The role of the growth environment for the tanshinone content was evaluated in five-week-old *S. miltiorrhiza* roots, leaves, and stems with and without *HMGR4* overexpression growing in soil in relation to the plant material grown in in vitro conditions.

The effect of GA_3_ (1 mg/L, 2.89 µM) or IAA (0.5 mg/L, 2.85 µM) on the production of tanshinones was estimated in five-week-old roots, leaves, and stems of *S. miltiorrhiza* overexpressing *HMGR4*, grown in vitro and treated with the hormones against untreated plant material.

The role of plant organ for the accumulation of tanshinones was assessed in five-week-old *S. miltiorrhiza* roots, leaves, and stems with and without *HMGR4* overexpression growing in soil and in in vitro conditions.

### 4.5. RNA Isolation, Reverse Transcription and Quantitative Real-Time PCR

Total RNA was isolated in accordance with the protocol given in NucleoSpin RNA Plant and Fungi kit (Macherey-Nagel, Duren, Germany). Plant material was ground under liquid nitrogen to a fine powder using mortar and pestle. The samples were digested by RNase-free rDNase (Macherey-Nagel, Duren, Germany) to assure removal of genomic DNA. Isolated RNA was stored at −80 °C. The concentration and purity of the RNA were evaluated using Nanophotometer P300 (Implen, Munich, Germany). The obtained A_260_/A_280_ ratios were within the range of 1.9–2.1 and A_260_/A_230_ ratios were ~2.

The reverse transcription reactions were carried out using Maxima H Minus Reverse Transcriptase, Oligo(dT)18 Primer, dNTP Mix, RiboLock RNase Inhibitor, and nuclease-free water (Thermo Scientific, Waltham, MA, USA) according to the manufacturer’s protocol. The quantity of RNA was adjusted to achieve the same final RNA concentration in a given experiment. No reverse transcriptase and no template controls were applied. The prepared cDNA was stored at −20 °C.

In order to investigate the expression of *HMGR4*, real-time PCR reactions were performed. *Actin* (*ACT7*) was used as a reference gene [65]. Gene-specific primers (Table 2) were created based on JN831103.1 and HM051058.1 sequences using Primer3web version 4.1.0 (https://primer3.ut.ee/, accessed on 5 June 2022) and Jellyfish version 1.5 tools. Expected sizes of *HMGR4* and *ACT7* fragments (Table 2) were confirmed by agarose gel electrophoresis. Amplification reactions were conducted using SYBR Green JumpStart Taq ReadyMix (Sigma-Aldrich, Saint Louis, MO, USA) according to the manufacturer’s instructions on Rotor-Gene 6000 (Corbett Research, Manchester, United Kingdom). The real-time PCR reaction parameters were as follows: initial denaturation (95 °C, 10 min), 40 cycles of denaturation (95 °C, 20 s), primer annealing (60 °C, 30 s), extension (72 °C, 10 s). The obtained products were melted in the temperature range of 72–95 °C with an increment of 1 °C. Ct values for *HMGR4* were normalised to Ct values for *ACT7* and calculated relative to a calibrator according to Pfaffl method [66].

### 4.6. Quantitative Analysis of Tanshinones

Roots, stems and leaves of *S. miltiorrhiza* were freeze-dried in a lyophiliser Alpha 1-2 LD (Martin Christ, Osterode am Harz, Germany) under 0.1 mbar pressure and ground with a pestle and mortar to a fine powder. The obtained powder (50 mg) was extracted with methanol (2 mL) under ultrasonic treatment for one hour at room temperature. The mixture was then centrifuged at 14,000× *g* for 5 min and then the supernatant was filtered through a 0.2 µm organic membrane filter (Millipore, Burlington, MA, USA) [67].

The tanshinone content was determined with a UHPLC 1290 Infinity instrument (Agilent Technologies, Santa Clara, CA, USA). Chromatographic separation was performed on a Zorbax Eclipse XDB-C18 column (3 mm × 100 mm, 1.8 µm particle size; Agilent Technologies, Santa Clara, CA, USA) with a Zorbax Eclipse XDB-C18 pre-column (3 mm × 5 mm, 1.8 µm particle size; Agilent Technologies, Santa Clara, CA, USA) thermostatted at 30 °C. The mobile phase consisted of 0.1% (*v/v*) formic acid acetonitrile solution (A) and 0.1% (*v/v*) formic acid aqueous solution (B), and the flow rate was 0.4 mL/min. The following gradient was used (all concentrations are *v/v*): 0–2 min, 40–55% A; 2–12 min, 55–50% A; 12–13 min, 50–80% A; 13–17 min, 80–95% A; 17–20 min, 95% A. The column and pre-column were equilibrated to 40% A for 1.5 min. The samples were injected in a volume of 1 µL and the wavelength of 270 nm was applied for the detection of tanshinones. HPLC grade DHTI, CT, TI, TIIA standards (Sigma-Aldrich, Saint Louis, MO, USA) were used for calibration. HPLC grade methanol, acetonitrile, and water were provided by Sigma-Aldrich (Saint Louis, MO, USA). More details on the UHPLC analysis are provided in Table 3. The data were collected and processed using ChemStation 3D software.

### 4.7. Statistical Analysis

Statistica 13.3 software (TIBCO Software Inc, Palo Alto, CA, USA) was used for analyses. The collected UHPLC results were checked for normal distribution using the Shapiro–Wilk test, and subsequent analyses were performed using the Kruskal–Wallis test, Mann–Whitney U test and *t*-test. Values with *p* < 0.05 were considered statistically significant. Expression values were given as mean ± SD.

## 5. Conclusions

The most important observation from the conducted research concerns the important role played by *HMGR4* in the biosynthesis of tanshinones, which is reflected in the content of DHTI, CT, TI, TIIA in the roots and TIIA in the stems and leaves with gene overexpression.

Other conclusions:

GA_3_, IAA and SA regulated the expression of the *S. miltiorrhiza HMGR4* gene, confirming the results of the in silico promoter analysis.

The soil environment promoted a higher accumulation of all tested metabolites in roots and TIIA in leaves compared to in vitro conditions. However, it is worth noting that the amounts of DHTI and TI in in vitro roots with *HMGR4* overexpression were higher than in soil-grown roots without overexpression.

Apart from the positive effect on the appearance of TIIA in the studied stems and leaves of *S. miltiorrhiza*, *HMGR4* overexpression did not change the characteristic organ-dependent pattern of tanshinone accumulation, i.e., the main source was the root, with trace amounts observed in stems and leaves.

GA_3_ increased CT and TIIA production in roots, while IAA reduced the biosynthesis of all tested metabolites.

The greatest efficiency of tanshinone biosynthesis was found to result from a combination of three traits, namely *HMGR4* gene overexpression, root organ, and cultivation in soil conditions.

Future research could investigate the mechanisms controlling *S. miltiorrhiza HMGR4* gene expression. TFs regulating *HMGR4* expression could be isolated using the yeast-one hybrid (Y1H) system and then functionally characterised [68]. The role of specific TFBSs in the response of *HMGR4* to abiotic or biotic factors could be verified by its mutagenesis [69]. TF networks that play a key role in the regulation of *HMGR4* gene expression could be explored through transcriptomic RNA sequencing and weighted gene co-expression network analysis (WGCNA) [70,71].

## Figures and Tables

**Table 1:** Potential transcription factors (TFs) that bind to the *Salvia miltiorrhiza HMGR4* promoter sequence and respond to gibberellic acid (GA_3_), indole-3-acetic acid (IAA), salicylic acid (SA) signals found using PlantPan 2.0 tool.

TF Family Name	TF Gene Name and Locus	TFBSs Number
**GA_3_**		
AT-Hook	*AHL25*; At4g35390	6
MYB-related	*CCA1*; At2g46830	1
	*RVE8*; At3g09600	2
	*RVE4*; At5g02840	
Homeodomain; HD-ZIP	*ATHB-23*; At1g26960	1
bHLH	*PIF3*; At1g09530	2
GATA	*GATA22*; At4g26150	62
	*GATA21*; At5g56860	
MADS box; MIKC	*SOC1*; At2g45660	8
	*AGL24*; At4g24540	15
MADS box	*AGL71*; At5g51870	13
	*AGL72*; At5g51860	14
	*AGL42*; At5g62165	
NF-YC	*NFYC3*; At1g54830	48
**IAA**		
MYB-related	*CCA1*; At2g46830	1
	*RVE 8*; At3g09600	2
Homeodomain; HD-ZIP	*HAT2*; At5g47370	2
	*ATHB-20*; At3g01220	3
CAMTA	*CAMTA1*; At5g09410	2
MADS box; MIKC	*AGL14*; At4g11880	17
**SA**		
NAC; NAM	*NAC081*; At5g08790	1
	*NAC062*; At3g49530	2
MYB	*MYB46*; At5g12870	4
Myb/SANT; MYB	*MYB3*; At1g22640	1
MYB-related	*CCA1*; At2g46830	1
	*RVE8*; At3g09600	2
	*RVE4*; At5g02840	
Dof	*DOF1.1*; At1g07640	34
	*DOF3.4*; At3g50410	
bZIP	*TGA2*; At5g06950	32
bHLH	*LRL1*; At2g24260	2
CG-1; CAMTA	*CAMTA2*; At5g64220	3
CAMTA	*CAMTA4*; At1g67310	2
	*CAMTA6*; At3g16940	
CSD	*CSP2*; At4g38680	2
WRKY	*WRKY6*; At1g62300	9
	*WRKY40*; At1g80840	
	*WRKY4*; At1g13960	8
	*WRKY60*; At2g25000	
	*WRKY21*; At2g30590	
	*WRKY54*; At2g40750	
	*WRKY70*; At3g56400	
	*WRKY53*; At4g23810	
	*WRKY18*; At4g31800	
	*WRKY26*; At5g07100	
	*WRKY38*; At5g22570	
	*WRKY30*; At5g24110	

**Table 2:** Primers used in the study.

Primer Name	Sequence	Product Size [bp]
**Confirmation of Transformation**		
Kanamycin_F	TGATCTCGTCGTGACCCAT	234
Kanamycin_R	AGAAGGCGATAGAAGGCGA	
**Real-Time PCR**		
HMGR4_F	CTCAACCTGCTTGGCGTAA	185
HMGR4_R	AGTCTCGTGATGTCCCTGCT	
ACT7_F	TCCGTCTTGATCTTGCTGGT	170
ACT7_R	CGTCTTTGCAGTTTCGAGCT	

**Table 3:** Details of UHPLC tanshinone analysis in *S. miltiorrhiza*.

Analyte	Retention Time [min]	Standard Curve	R^2^
DHTI	6.025	y = 190.163x − 16.690	0.99973
CT	10.009	y = 146.106x − 10.926	0.99992
TI	10.938	y = 241.276x − 21.816	0.99989
TIIA	14.808	y = 327.209x − 20.014	0.99996

## Data Availability

The data presented in this study are included in the article.

## Supplementary Materials

The following supporting information can be downloaded at: https://www.mdpi.com/article/10.3390/molecules27144354/s1, Table S1: Potential transcription factor binding sites (TFBSs) and interacting transcription factors (TFs) that respond to gibberellic acid (GA_3_) signals found in *Salvia miltiorrhiza HMGR4* promoter sequence using PlantPan 2.0 tool; Table S2: Potential TFBSs and interacting TFs that respond to indole-3-acetic acid (IAA) signals found in *S. miltiorrhiza HMGR4* promoter sequence using PlantPan 2.0 tool; Table S3: Potential TFBSs and interacting TFs that respond to salicylic acid (SA) signals found in *S. miltiorrhiza HMGR4* promoter sequence using PlantPan 2.0 tool.

## Sample Availability

Samples of the compounds are not available from the authors.
