# Supplementary material for: The Expression Profiles of the Salvia miltiorrhiza 3-Hydroxy-3-methylglutaryl-coenzyme A Reductase 4 Gene and Its Influence on the Biosynthesis of Tanshinones
Source: Molecules. 2022 Jul 7;27(14):4354. doi: 10.3390/molecules27144354 (PMC9317829; doi:10.3390/molecules27144354)
Supplement: Supplementary file 1 [file molecules-27-04354-s001.zip › Table S1.pdf]

Table S1. Potential transcription factor binding sites (TFBSs) and interacting transcription factors (TFs) that respond to gibberellic acid (GA_3) signals found in *Salvia miltiorrhiza HMGR4* promoter sequence using PlantPan 2.0 tool.

**TFBS motif and localisation**[a,b,c]		**TF family name**	**TF gene name and locus**	**processes in which TF is involved**
**AATTA 1268**<br>**AATAT 1263**	AATAA 309<br>TTATT 475<br>ATATT 345; 631	AT-Hook	*AHL25;*<br>At4g35390	negative regulation of GA_3-mediated signaling pathway; vegetative to reproductive phase transition of meristem
TATCT 186		MYB-related	*CCA1;*<br>At2g46830	responded e.g. to GA_3; negative regulation of circadian rhythm; phytochrome regulation; long-day photoperiodism and flowering; regulation of protein homodimerization activity
ATATC 1166	**GATAT 1215**		*RVE8;*<br>At3g09600	responded e.g. to GA_3; histone H3 acetylation; photoperiodism and flowering; positive regulation of circadian rhythm
			RVE4;<br>At5g02840	responded e.g. to GA_3; regulation of circadian rhythm
ATAAT 309		Homeodomain;<br>HD-ZIP	*ATHB-23;*<br>At1g26960	induced by GA_3; lateral root development
CACAT 644	ACATG 644	bHLH	*PIF3;*<br>At1g09530	regulation of GA_3-mediated, phytochrome and red/far-red light signaling pathways; de-etiolation; positive regulation of anthocyanin metabolic process
GGATC 220<br>TATCC 377<br>GGATA 392<br>GATAC 393<br>GATAA 439<br>**CGATG 1227**<br>**AGATT 1405**<br>CGATT 424; 1143<br>TATCG 448; 1169<br>CATCT 753; 872<br>**AATCA** 11; **1315**<br>AATCG 75; 130<br>GATCG 101; 221	GGATG 962; 1178<br>ATATC 189; 1168<br>GTATC 376; 761<br>CTATC 447; 891<br>**GATAT** 769; **1217**<br>AATCT 15; 197; 919<br>**AGATG** 120; 662; **1427**<br>CATCC 141; 778; 999<br>TATCT 162; 190; 326<br>AATCC 179; 204; 548<br>**TGATA** 438; 768; **1216**<br>**TTATC** 161; 325; 689; 860; **1226**<br>**TATCA** 690; 762; 861; 892; **1213**<br>TGATT 648; 1076; 1101; 1131; 1198	GATA	*GATA22;*<br>At4g26150<br><br>*GATA21;*<br>At5g56860	induced e.g. by GA_3; modulation of chloroplast development, growth and division in cytokinin-dependent manner; chlorophyll biosynthesis; regulation of sugar-sensing genes; circadian rhythm; regulation of germination, senescence, elongation growth and flowering time; influence leaf starch content
TTTTT 77; 78; 79; 80; 81; 82; 83; 84		MADS box;	*SOC1;*	up-regulated by GA_3; integration of signals from photoperiod,

		MIKC	At2g45660	vernalisation and autonomous floral induction pathways; when associated with AGL24, mediate effect of GA_3 on flowering under short-day conditions
AATGG 842<br>TTTTG 805<br>C**AAAA 1396**	ATTTCC 804; 801<br>**GGAAA 1364; 1362**<br>TTTTT 77; 78; 79; 80; 81; 82; 83; 84		*AGL24;*<br>At4g24540	up-regulated by GA_3; floral transition in response to vernalization; effect of GA_3 on flowering under short-day conditions; identity of floral meristem; inflorescence fate in apical meristems
AATGG 842<br>TTTCC 804; 801	**GGAAA 1364; 1362**<br>TTTTT 77; 78; 79; 80; 81; 82; 83; 84	MADS box	*AGL71;*<br>At5g51870	act through GA_3-dependent pathway; flowering time; flowering at shoot apical and axillary meristems
TTTTG 805<br>**CAAAA 1396**	TTTCC 804; 801<br>**GGAAA 1364; 1362**<br>TTTTT 77; 78; 79; 80; 81; 82; 83; 84		*AGL72;*<br>At5g51860	
			AGL42;<br>At5g62165	act through GA_3-dependent pathway; flowering time; flowering at shoot apical and axillary meristems; control of flower organ senescence and abscission; leaf senescence
CTAAT 42<br>CCCAT 139<br>GTTGG 389<br>CCAAC 530<br>AGTGG 592<br>TTTGG 811<br>CCTAT 890<br>ATGGG959<br>ATTGC 1039<br>CGAAT 1115<br>**ATTAG 1389**<br>CCAAT 195; 202	ATCGG 102; 131<br>CCAAG 149; 213<br>AATGG 462; 847<br>ATTGA 766; 1099<br>CCATT 169; 266; 701<br>**CCACT** 144; 380; **1364**<br>**TCAAT** 13; 1149; **1253**<br>**ACAAT** 460; 800; **1247**<br>ATTGT 599; 637; 1145; 1152<br>**CCAAA** 176; 534; **1261; 1396**<br>**CAAAT** 177; 469; 897; 1107; 1161**;**<br>**1262; 1313**	NF-YC	*NFYC3;*<br>At1g54830	involved in GA_3- and abscisic acid-activated signaling pathway; long-day photoperiodism and flowering; positive regulation of photomorphogenesis; regulation of seed germination

[a] For TFBSs only most conserved positions within a matrix were listed. [b] Binding sites localised in proximal promoter region are in bold. [c] The transcription start site (TSS) is located at 1500 nucleotide of the studied promoter sequence.
