# Supplementary material for: The Expression Profiles of the Salvia miltiorrhiza 3-Hydroxy-3-methylglutaryl-coenzyme A Reductase 4 Gene and Its Influence on the Biosynthesis of Tanshinones
Source: Molecules. 2022 Jul 7;27(14):4354. doi: 10.3390/molecules27144354 (PMC9317829; doi:10.3390/molecules27144354)
Supplement: Supplementary file 1 [file molecules-27-04354-s001.zip › Table S3.pdf]

Table S3. Potential TFBSs and interacting TFs that respond to salicylic acid (SA) signals found in *S. miltiorrhiza HMGR4* promoter sequence using PlantPan 2.0 tool.

**TFBS motif and localisation[a,b,c]**		**TF family name**	**TF gene name and locus**	**processes in which TF is involved**
CGTA 720		NAC; NAM	*NAC081;* At5g08790	induced e.g. by SA; regulation of host basal defense responses against viral infection; repression of pathogenesis-related proteins; suppression of brassinosteroid-inactivating enzymes CYP734A1/BAS1 and CYP72C1/SOB7; integration of brassinosteroid homeostasis and seedling development; spatial regulation of brassinosteroid homeostasis, regulation of hypocotyl elongation and root growth by suppression of brassinosteroid catabolism; transactivation of auxin biosynthetic gene *NIT2* promoter; abscisic acid-inducible leaf senescence signalling; seed development and morphology
AGTAA 124	AGTAA 565		*NAC062;* At3g49530	induced e.g. by SA; induction of pathogenesis-related genes in response to cold; regulatory role in -mediated drought-resistance response
ACCTA 887	**AACCA** 152; 526; **1485**	MYB	*MYB46;* At5g12870	slightly induced by SA; positive regulation of secondary wall biosynthesis in fibers and vessels; defense response to fungi; activation of mannan synthase *CSLA9* gene
ACCTA 886		Myb/SANT; MYB	*MYB3;* At1g22640	induced e.g. by SA; cell differentiation; cinnamic acid biosynthetic process; negative regulation of metabolic process
TATCT 186		MYB-related	*CCA1;* At2g46830	responded e.g. to SA; see also *CCA1* in GA$_3$ part
ATATC 1166	**GATAT 1215**		*RVE8;* At3g09600	responded e.g. to SA; see also *RVE8* in GA$_3$ part
			RVE4; At5g02840	responded e.g. to SA; see also *RVE4* in GA$_3$ part
GCCTT 79<br>GCTTT 369<br>AAGGG 1031<br>**AAGGC 1290**	ACCTT 158; 184; 605<br>TCTTT 252; 586; 874<br>AAAGC 400; 571<br>**AAAGA** 939; **1413; 1420**	Dof	*DOF1.1;* At1g07640	induced e.g. by SA; regulation of root development and glucosinolate biosynthetic process; promotion of radial growth of protophloem sieve elements and apical dominance

A**AGGA 1372**<br>CCTTT 80; 807<br>AAAGT 109; 982	**AAAGG** 1030; **1289; 1371**<br>TCCTT 275; 328; 338; 806; 1156<br>**ACTTT** 382; 881; 509; 612; 1021; **1297**		*DOF3.4;*<br>At3g50410	induced e.g. by SA; cell wall modification; positive regulation of cell cycle; zinc ion binding
CGTCC 362<br>GGACG 407<br>CGTTA 687<br>CGTAA 722<br>TGACA 796<br>GTCAT 944<br>TCACG 1112<br>**CCTCA 1458**	TGACT 5; 832<br>ATGAC 4; 795<br>**AGTCA** 111; **1240**<br>CGTCA 232; 229<br>CGTCT 250; 782<br>TGAAG 565; 789; 969<br>**TGACG 1210; 1208**<br>CTTCA 237; 607; 955; 1158<br>**TGTCA** 601; 913; 948; 1147; **1251**	bZIP	*TGA2;*<br>At5g06950	recognition and binding to as-1-like cis elements mediating auxin- and SA-inducible transcription; induction of systemic acquired resistance via regulation of pathogenesis-related genes expression
CCATG 574	GCACA 643	bHLH	*LRL1;*<br>At2g24260	induced e.g. by SA; microgametogenesis; pollen sperm cell differentiation; root hair cell development; root hair cell differentiation and elongation
CCGCG 229	CGCGT 226; 229	CG-1; CAMTA	*CAMTA2;*<br>At5g64220	induced e.g. by SA; regulation of transcriptional activity in response to calcium signals; binding calmodulin in calcium-dependent manner; freezing tolerance; positive regulation of general stress response; tolerance to aluminium
CCGCG 229	CGCGT 229	CAMTA	*CAMTA4;*<br>At1g67310	induced e.g. by SA; regulation of transcriptional activity in response to calcium signals; binding to calmodulin in calcium-dependent manner; positive regulation of general stress response
			CAMTA6;<br>At3g16940	induced e.g. by SA; binding of calmodulin in calcium-dependent manner; regulation of transcriptional activity in response to calcium signals
TTTAT 62; 667		CSD	*CSP2;*<br>At4g38680	induced e.g. by SA; chaperone that unwind nucleic acid duplex; acceleration of seed germination and seedling growth under cold stress; enhancement of freezing tolerance; flowering transition; flower and seed development; seed germination under salt stress regulation of respiratory oxygen uptake
TGACT 5; 832<br>TTGAC 830; 831	**AGTCA** 111; **1240**<br>**GTCAA** 913; 1147; **1251**	WRKY	*WRKY6;*<br>At1g62300	induced e.g. by SA; control of processes related to senescence and pathogen defense; cellular response to boron-containing substance deprivation; response to chitin; cellular response to

			phosphate starvation; modulation of phosphate homeostasis and Pi translocation
		WRKY40; At1g80840	responded to SA; defense response to bacterium, fungus; response to chitin and wounding
TTGAC 831<br>TGACT 5; 832	**AGTCA** 111; **1240**<br>**GTCAA** 913; 1147; **1251**	*WRKY4;* At1g13960	induced e.g. by SA; positive role in resistance to necrotrophic pathogens (e.g. *Botrytis cinerea*); negative effect on plant resistance to biotrophic pathogens (e.g. *Pseudomonas syringae*)
		WRKY60; At2g25000	responded to SA; regulation of: defense response to bacterium and fungus
		WRKY21; At2g30590	induced by SA; glucosinolate metabolic process; calmodulin binding
		WRKY54; At2g40750	induced e.g. by SA; negative regulation of leaf senescence; prevention of stomatal closure and, consequently, osmotic stress tolerance; promotion of brassinosteroid-regulated plant growth; prevention of defense response to necrotrophic pathogens *Pectobacterium carotovorum* and *B. cinerea*; promotion of defense against biotrophic/hemibiotrophic pathogens *Pseudomonas syringae* pv. tomato
		WRKY70; At3g56400	responded e.g. to SA; senescence; biotic and abiotic stress responses by modulation of various phytohormones signaling pathways; positive regulation of SA-mediated signal pathway; negative regulation of jasmonic acid-mediated signal pathway; prevention of defense response to necrotrophic pathogens *P. carotovorum* and *B. cinerea*; promotion of defense responses against biotrophic/hemibiotrophic SA-monitored pathogens (e.g. *P. syringae*, *Erwinia carotovora* subsp. *carotovora* and *Erysiphe cichoracearum*); defense response toward insects (e.g. *Plutella xylostella* and *Brevicoryne brassicae*); repression of biosynthesis of phytoalexin, camalexin, indol-3-ylmethyl glucosinolate and SA; promotion of brassinosteroid-regulated plant growth; prevention of stomatal closure and, consequently, osmotic stress tolerance; cellular response to hypoxia
		WRKY53; At4g23810	induced by SA; regulation of early events of leaf senescence; regulation of jasmonic acid-mediated signaling pathway; defense response to bacterium; response to chitin

	WRKY18; At4g31800	induced e.g. by SA; regulation of defense response to bacterium and fungus; response to chitin
	WRKY26; At5g07100	induced e.g. by SA; positive regulator of plant thermotolerance by participating in ethylene-response signal transduction pathway
	WRKY38; At5g22570	involved in SA mediated signaling pathway; defense response to bacterium
	WRKY30; At5g24110	induced by SA; leaf senescence
	WRKY8; At5g46350	induced e.g. by SA; positive regulator of salt stress response; negative regulation of basal resistance to bacterial pathogen *P. syringae*; positive regulation of resistance to fungal pathogen *B. cinerea*; positive regulation of defense response againt tobamovirus (TMV)

[a] For TFBSs only most conserved positions within a matrix were listed. [b] Binding sites localised in proximal promoter region are in bold. [c] The TSS is located at 1500 nucleotide of the studied promoter sequence.
