# Supplementary material for: The Expression Profiles of the Salvia miltiorrhiza 3-Hydroxy-3-methylglutaryl-coenzyme A Reductase 4 Gene and Its Influence on the Biosynthesis of Tanshinones
Source: Molecules. 2022 Jul 7;27(14):4354. doi: 10.3390/molecules27144354 (PMC9317829; doi:10.3390/molecules27144354)
Supplement: Supplementary file 1 [file molecules-27-04354-s001.zip › Table S2.pdf]

Table S2. Potential TFBSs and interacting TFs that respond to indole-3-acetic acid (IAA) signals found in *S. miltiorrhiza HMGR4* promoter sequence using PlantPan 2.0 tool.

TFBS motif and localisation[a,b,c]		TF family name	TF gene name and locus	processes in which TF is involved
TATCT 186		MYB-related	*CCA1;* At2g46830	responded e.g. to IAA; see also *CCA1* in GA_3 part
ATATC 1166	**GATAT 1215**	MYB-related	*RVE 8;* At3g09600	responded e.g. to IAA; see also *RVE8* in GA_3 part
TGATT 645; 1195		Homeodomain; HD-ZIP	*HAT2;* At5g47370	induced by IAA; auxin-mediated morphogenesis; negative regulation of lateral root elongation
ATAAT 310	AATAA 308; 307	Homeodomain; HD-ZIP	*ATHB-20;* At3g01220	induced e.g. by IAA; seed germination in micropylar endosperm
CCGCG 229	CGCGT 229	CAMTA	*CAMTA1;* At5g09410	IAA signaling and response to abiotic stresses; binding of calmodulin in calcium-dependent manner; freezing tolerance; drought stress response
AATGG 842<br>TTTTG 805<br>**CAAAA 1396**	TTTCC 804; 801<br>A**GAAA** 293; **1402**<br>**GGAAA 1364; 1362**<br>TTTTT 77; 78; 79; 80; 81; 82; 83; 84	MADS box; MIKC	*AGL14;* At4g11880	induced by IAA; regulation of root development by controlling meristem size and patterning of root apical meristem; regulation of auxin transport and gradients in root meristematic cells; regulation of shoot apical meristem cell identities and transitions; flowering transition, flower meristem maintenance and determinacy; plants over-expressing *AGL14* show early flowering phenotype and flowers have vegetative traits

[a] For TFBSs only most conserved positions within a matrix were listed. [b] Binding sites localised in proximal promoter region are in bold. [c] The TSS is located at 1500 nucleotide of the studied promoter sequence.
